# LGR5 and BMI1 Increase Pig Intestinal Epithelial Cell Proliferation by Stimulating WNT/β-Catenin Signaling

**DOI:** 10.3390/ijms19041036

**Published:** 2018-03-30

**Authors:** Xiang-Guang Li, Zhe Wang, Rong-Qiang Chen, Hou-Long Fu, Chun-Qi Gao, Hui-Chao Yan, Guang-Xu Xing, Xiu-Qi Wang

**Affiliations:** 1 College of Animal Science, South China Agricultural University/Guangdong Provincial Key Laboratory of Animal Nutrition Control/National Engineering Research Center for Breeding Swine Industry, Guangzhou 510642, China; xgli@gdut.edu.cn (X.-G.L.); chen_rong_qiang@yeah.net (R.-Q.C.); fuhoulong@126.com (H.-L.F.); cqgao@scau.edu.cn (C.-Q.G.); yanhc@scau.edu.cn (H.-C.Y.); 2 Department of Pharmaceutical Engineering, School of Chemical Engineering and Light Industry, Guangdong University of Technology, Guangzhou 510006, China; 3 Tianhe Foreign Language School, Guangzhou 510627, China; wangzhe1923@163.com; 4 Key Laboratory of Animal Immunology of the Ministry of Agriculture, Henan Academy of Agricultural Sciences, Zhengzhou 450002, China; xingguangxu@163.com

**Keywords:** LGR5, BMI1, WNT/β-catenin signaling, cell proliferation, intestinal epithelial cells

## Abstract

Leucine-rich repeat-containing G protein-coupled receptor 5 (LGR5) and B-cell-specific Moloney murine leukemia virus insertion site 1 (BMI1) are markers of fast-cycling and quiescent intestinal stem cells, respectively. To determine the functions of these proteins in large animals, we investigated their effects on the proliferation of intestinal epithelial cells from pigs. Our results indicated that LGR5 and BMI1 are highly conserved proteins and that the pig proteins have greater homology with the human proteins than do mouse proteins. Overexpression of either *LGR5* or *BMI1* promoted cell proliferation and WNT/β-catenin signaling in pig intestinal epithelial cells (IPEC-J2). Moreover, the activation of WNT/β-catenin signaling by recombinant human WNT3A protein increased cell proliferation and LGR5 and BMI1 protein levels. Conversely, inhibition of WNT/β-catenin signaling using XAV939 reduced cell proliferation and LGR5 and BMI1 protein levels. This is the first report that LGR5 and BMI1 can increase proliferation of pig intestinal epithelial cells by activating WNT/β-catenin signaling.

## 1. Introduction

The intestinal epithelium, which is covered by a monolayer of epithelial cells, completely renews itself every four or five days, driven by intestinal stem cells (ISCs) [1]. Two populations of stem cells—fast-cycling stem cells known as crypt base columnar (CBC) cells, and quiescent stem cells known as +4 stem cells—have been found in the intestinal crypts [2,3]. Leucine-rich repeat-containing G protein-coupled receptor 5 (LGR5) is a surface molecular marker used to accurately locate and isolate pure CBC cells [2,4], whereas B-cell-specific Moloney murine leukemia virus insertion site 1 (BMI1) is a marker of +4 stem cells [5]. Yan et al. [6] demonstrated that BMI1-marked quiescent ISCs can give rise to fast-cycling ISCs under stress conditions. Knocking out LGR5 in mice resulted in neonatal lethality by causing gastrointestinal distension [7] or precocious Paneth cell differentiation [8]. However, the coding sequence of pig LGR5 has not been obtained, and the effects of LGR5 and BMI1 on the proliferation of intestinal epithelial cells from large animals remain unknown.

WNT/β-catenin signaling is critical for the proliferation of intestinal epithelial cells and the maintenance of ISCs [9]. This signaling pathway facilitates intestinal renewal by stimulating cell proliferation and differentiation [10,11,12]. In the absence of WNT, a β-catenin degradation complex consisting of AXIN, adenomatous polyposis coli (APC) and glycogen synthase kinase 3β (GSK3β) binds to and phosphorylates β-catenin, which is then degraded. When the binding of WNT to its receptors induces the construction of the degradation complex, β-catenin becomes stabilized and subsequently binds to T-cell factor (TCF) in the nucleus to upregulate target genes [13].

LGR5 was found to be not only a target gene but also a potentiating receptor upstream of the WNT/β-catenin signaling pathway [14]. Therefore, we hypothesized that the ISC markers LGR5 and BMI1 promote WNT/β-catenin signaling to stimulate the proliferation of pig intestinal epithelial cells.

## 2. Results

### 2.1. Cloning of Pig LGR5

**Figure 1:**
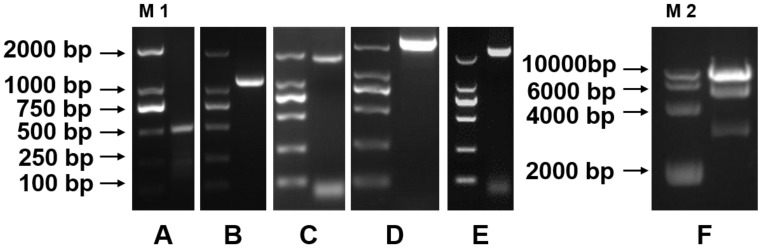
The cloning of pig *LGR5* (**A**–**D**) and the identification of the recombinant plasmid *LGR5*-pcDNA3.1 (**E**,**F**). M 1: DNA Marker 2000. M 2: DNA Marker 10,000. (**A**) 3′ end fragment; (**B**) *LGR5* A fragment; (**C**) *LGR5* B fragment; (**D**) *LGR5* ORF; (**E**) PCR identification of the recombinant plasmid *LGR5*-pcDNA3.1; (**F**) enzyme-digesting identification of the recombinant plasmid *LGR5*-pcDNA3.1 by *Bam*HI and *Xba*I.

**Figure 2:**
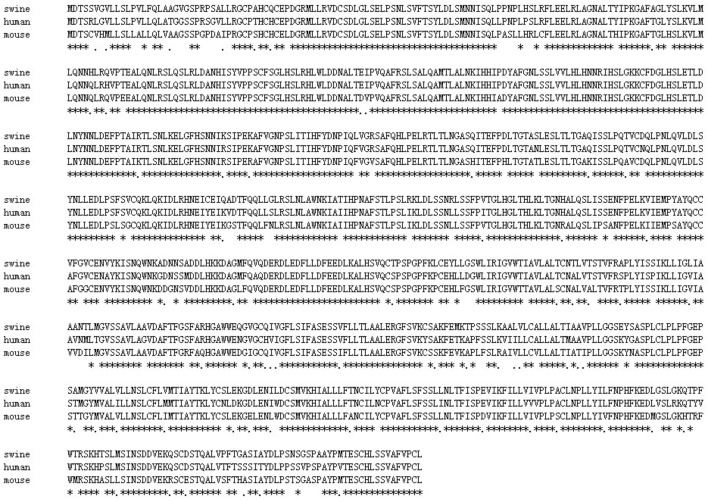
Comparison of the mouse, human and pig LGR5 protein sequences. The pig LGR5 protein was predicted from the cloned nucleotide sequence via DNASTAR (www.dnastar.com), and the comparison was conducted with the same software. * indicates the same amino acid residue.

To obtain pig *LGR5* cDNA, we designed specific primers for PCR amplification based on the conserved human and mouse sequences (Table S1). We obtained the complete pig *LGR5* cDNA (GenBank ID: KP717080.1), which is 2832 base pairs (bp) long and contains a 2724-bp open reading frame (ORF) and a 108-bp 3′ untranslated region (Figure 1). The homology of the pig *LGR5* coding sequence with the human sequence was found to be 89.65%, while the protein homology was 90.30% (Figure 2). The LGR5 protein contains seven transmembrane domains and is most likely located in the cytomembrane. Bioinformatics performed with DNASTAR (www.dnastar.com) revealed that the signal peptide of the pig LGR5 protein is MDTSSVGVLLSLPVLFQLAAG. The *LGR5* overexpression vector was verified by reverse transcription-PCR (Figure 1E) and identified through enzyme digestion (Figure 1F).

### 2.2. LGR5 Overexpression Promotes Cell Proliferation and WNT/β-Catenin Signaling

**Figure 3:**
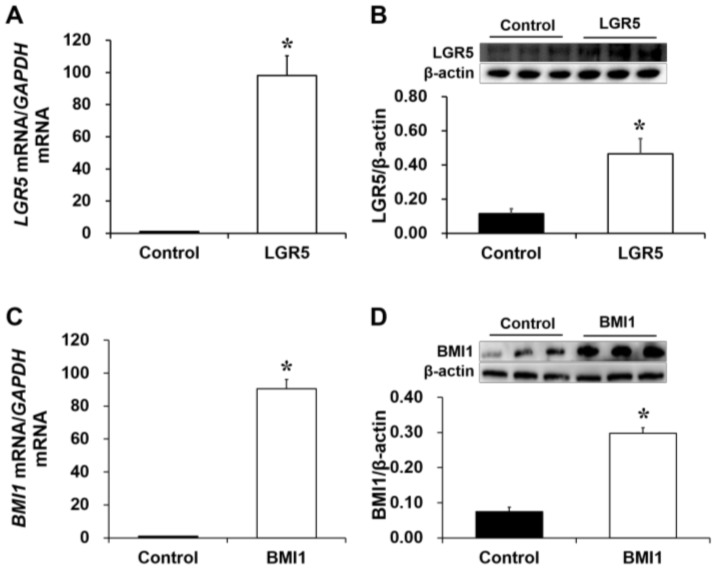
The expression of *LGR5* and *BMI1* in IPEC-2 cells. Identification of the *LGR5* mRNA abundance (**A**; *n* = 12) and protein level (**B**; *n* = 3) in the control and *LGR5*-overexpressing cell strains; Identification of the *BMI1* mRNA abundance (**C**; *n* = 12) and protein level (**D**; *n* = 3) in the control and *BMI1*-overexpressing cell strains. The results were confirmed by three independent experiments per treatment. Representative results of the three independent experiments are shown. The bars are the means ± SE, * indicates a significant difference (*p* < 0.05).

**Figure 4:**
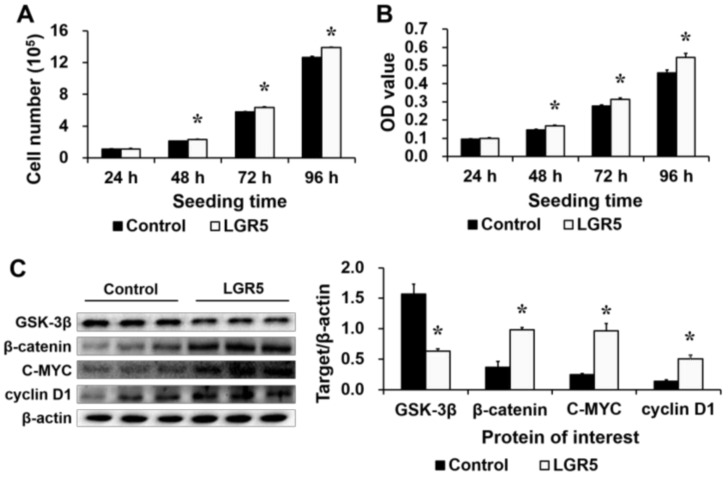
The effects of *LGR5* overexpression on cell proliferation and WNT/β-catenin signaling-related protein expression in IPEC-J2 cells. (**A**) The cell number was higher in the *LGR5*-overexpressing group than in the control group at 48, 72 and 96 h after seeding, as assessed by the cell count assay (*n* = 3); (**B**) The optical density (OD) value was higher in the *LGR5*-overexpressing group than in the control group at 48, 72 and 96 h after seeding, as assessed by the MTT assay (*n* = 20); and (**C**) The levels of WNT/β-catenin signaling-related proteins were assessed by Western blot (*n* = 3). The results were confirmed by three independent experiments per treatment. Representative results of the three independent experiments are shown. The bars are the means ± SE, * indicates a significant difference (*p* < 0.05).

Both *LGR5* mRNA (Figure 3A) and protein (Figure 3B) levels were much greater (*p* < 0.05) in *LGR5*-pcDNA3.1-transfected (LGR5) cells than in basic-pcDNA3.1-transfected (control) cells. Cell count and MTT assays on IPEC-J2 pig intestinal epithelial cells indicated that *LGR5* overexpression increased (*p* < 0.05) the cell numbers (Figure 4A) and optical density (OD) values (Figure 4B) at 48, 72 and 96 h after seeding. Furthermore, the protein levels of β-catenin, C-MYC and cyclin D1 were greater (*p* < 0.05) in *LGR5*-overexpressing cells than in control cells, whereas the protein level of GSK-3β was significantly lower (*p* < 0.05) in *LGR5*-overexpressing cells (Figure 4C). In short, pig LGR5 promoted cell proliferation and WNT/β-catenin signaling in IPEC-J2 cells.

### 2.3. BMI1 Overexpression Promotes Cell Proliferation and WNT/β-Catenin Signaling

**Figure 5:**
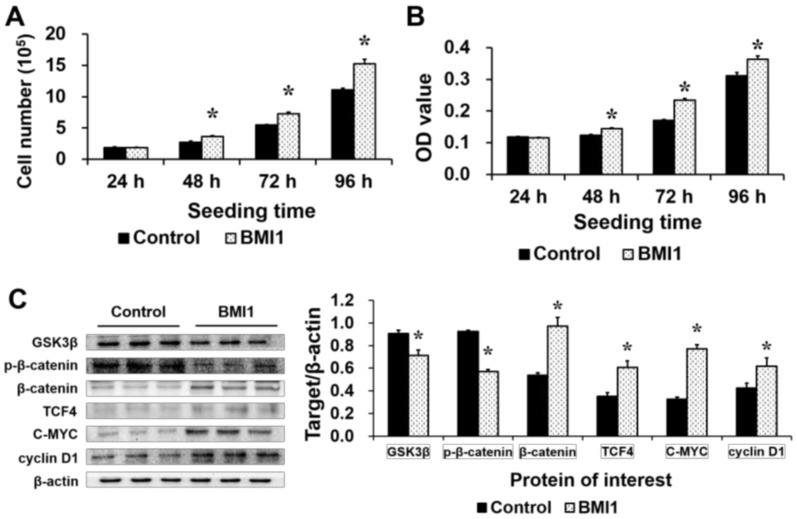
The effects of *BMI1* overexpression on cell proliferation and WNT/β-catenin signaling-related protein expression in IPEC-J2 cells. (**A**) The cell number was higher in the *BMI1*-overexpressing group than in the control group at 48, 72 and 96 h after seeding, as assessed by the cell count assay (*n* = 3); (**B**) The OD value was higher in the *BMI1*-overexpressing group than in the control group at 48, 72 and 96 h after seeding, as assessed by the MTT assay (*n* = 20); and (**C**) The levels of WNT/β-catenin signaling-related proteins were assessed by Western blot (*n* = 3). The results were confirmed by three independent experiments per treatment. Representative results of the three independent experiments are shown. The bars are the means ± SE, * indicates a significant difference (*p* < 0.05).

IPEC-J2 cells were transfected with *BMI1*-pcDNA3.1 plasmids, which were constructed previously in our lab to generate *BMI1*-overexpressing cells [15]. Both *BMI1* mRNA (Figure 3C) and protein (Figure 3D) levels were greater in *BMI1*-pcDNA3.1-transfected (BMI1) cells than in basic-pcDNA3.1-transfected (control) cells. Cell count and MTT assays demonstrated that *BMI1* overexpression increased the cell numbers (Figure 5A) and OD values (Figure 5B) at 48, 72 and 96 h after seeding. Additionally, *BMI1* overexpression reduced the protein levels of GSK3β and phospho-β-catenin (Ser33), but increased the expression of β-catenin, TCF4, C-MYC and cyclin D1 (Figure 5C) relative to the control group. Thus, BMI1 promoted cell proliferation and WNT/β-catenin signaling in IPEC-J2 cells.

### 2.4. WNT/β-Catenin Signaling Activation Increases Cell Proliferation and LGR5 and BMI1 Expression

**Figure 6:**
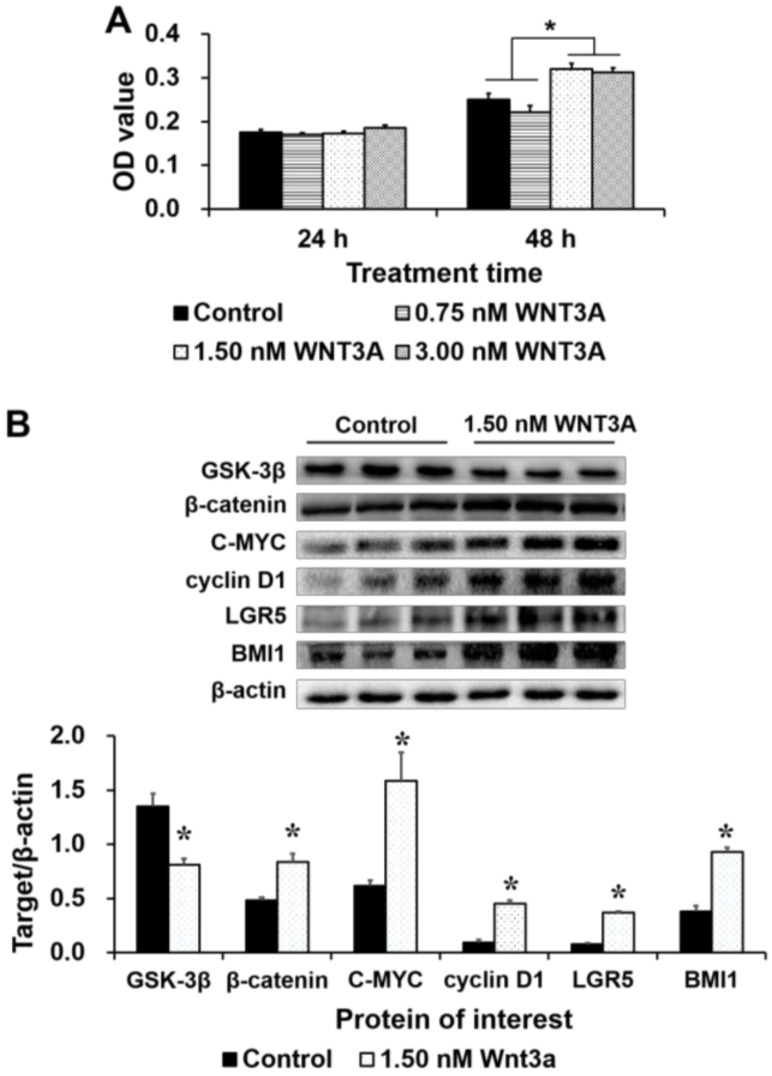
The effects of rhWNT3A protein supplementation on cell proliferation and protein expression in IPEC-J2 cells. (**A**) The OD values were higher in the 1.5- and 3.0-nmol/L WNT3A groups than in the control group at 48 h after treatment, as assessed by the MTT assay (*n* = 20); and (**B**) The levels of WNT/β-catenin signaling-related proteins, LGR5 and BMI1 were assessed by Western blot (*n* = 3). The results were confirmed by three independent experiments per treatment. Representative results of the three independent experiments are shown. The bars are the means ± SE, * indicates a significant difference (*p* < 0.05).

Recombinant human (rh) WNT3A protein was added to the growth medium at a final concentration of 0 (control), 0.75, 1.5 or 3.0 nmol/L for 24 or 48 h to activate WNT/β-catenin signaling in IPEC-J2 cells. In MTT assays, the OD values were significantly greater in cells treated with 1.5 and 3.0 nmol/L WNT3A for 48 h than in control cells (Figure 6A). Therefore, 1.5 nmol/L WNT3A was used for further experiments. At this concentration, rhWNT3A supplementation reduced the protein expression of GSK-3β, but increased the levels of β-catenin, C-MYC, cyclin D1, LGR5 and BMI1 (Figure 6B). Taken together, these results indicate that rhWNT3A supplementation activated WNT/β-catenin signaling, which subsequently increased cell proliferation and LGR5 and BMI1 expression.

### 2.5. WNT/β-Catenin Signaling Inhibition Suppresses Cell Proliferation and LGR5 and BMI1 Expression

**Figure 7:**
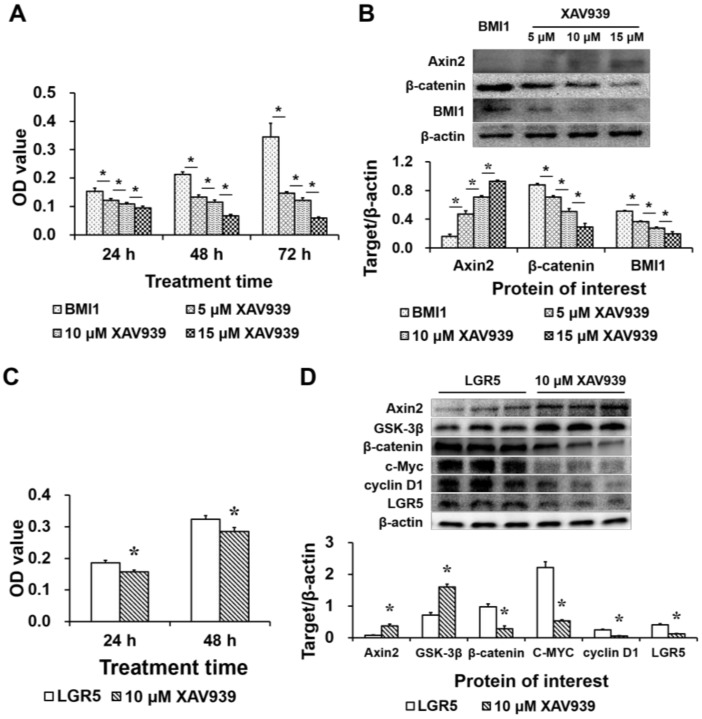
The effects of XAV939 supplementation on cell proliferation and protein expression in *LGR5*-overexpressing or *BMI1*-overexpressing IPEC-J2 cells. (**A**) The OD values were lower in the 5-, 10- or 15-μmol/L XAV939 groups than in the *BMI1*-overexpressing group at 24, 48 and 72 h after treatment, as assessed by the MTT assay (*n* = 20); (**B**) The levels of AXIN2, β-catenin and BMI1 were assessed by Western blot (*n* = 3); (**C**) The OD value was lower in the 10-μmol/L XAV939 group than in the *LGR5*-overexpressing group at 24 and 48 h after treatment, as assessed by the MTT assay (*n* = 20); and (**D**) The levels of WNT/β-catenin signaling-related proteins and LGR5 were assessed by Western blot (*n* = 3). The results were confirmed by three independent experiments per treatment. Representative results of the three independent experiments are shown. The bars are the means ± SE, * indicates a significant difference (*p* < 0.05).

We next treated cells with XAV939, a WNT/β-catenin signaling inhibitor that selectively stabilizes AXIN2 [16] and explored its effects on cell proliferation. Considering the very low abundance of *LGR5* and *BMI1* in wild-type IPEC-J2 cells, we used *LGR5*-overexpressing and *BMI1*-overexpressing cells for these experiments. First, XAV939 was added to the growth medium of *BMI1*-overexpressing IPEC-J2 cells at a final concentration of 0 (control), 5, 10 or 15 μmol/L. As expected, XAV939 supplementation reduced the OD values in MTT assays at 24, 48 and 72 h after treatment (Figure 7A) and reduced the expression of β-catenin and BMI1 (Figure 7B) dose-dependently. On the other hand, AXIN2 expression was greater in the XAV939-treated groups than in the *BMI1*-overexpressing group (Figure 7B).

Second, XAV939 at a final concentration of 0 (control) or 10 μmol/L was added to the growth medium of *LGR5*-overexpressing cells. XAV939 supplementation reduced cell proliferation (Figure 7C) and β-catenin, C-MYC, cyclin D1 and LGR5 expression (Figure 7D), but increased the protein levels of AXIN2 and GSK-3β (Figure 7D). In summary, XAV939 supplementation suppressed WNT/β-catenin signaling, LGR5 and BMI1 expression and cell proliferation.

## 3. Discussion

Due to the lack of effective molecular markers, ISCs are difficult to position and sort. Several molecular markers, including Musashi-1 [17], phospho-PTEN (phosphatase and tensin homolog) [18], Doublecortin- and Calmodulin Kinase-Like 1 and Eph receptors [19], have been considered as candidate markers for ISCs, but were later found not to identify cells with the two essential features of adult tissue stem cells—longevity and multipotency. However, LGR5 is a robust marker of CBC cells [1,6,20]. BMI1^+^ cells have been reported to be quiescent ISCs previously [6,21,22], and mature enteroendocrine cells more recently [20]. Considering that BMI^+^ cells are capable of self-renewal, are pluripotent and are required for crypt maintenance in vivo, and are able to form enteroids in vitro [3,6,23], it is difficult to deny the ISC characteristics of BMI^+^ cells.

Generally, LGR5 and BMI1 serve as markers of fast-cycling and quiescent ISCs, respectively. However, the functional properties of LGR5 and BMI1 in the intestinal epithelial cells of large animals have not been well documented. In the present study, the full-length coding sequence of pig *LGR5* was cloned. Bioinformatics analysis indicated that pig LGR5 is a membrane protein with seven transmembrane domains and a signal peptide consisting of 21 amino acids in the *N*-terminal region. These characteristics are similar to those of the human LGR5 protein [24]. Evolutionary tree analyses from the present study and our previous studies indicated that LGR5 and BMI1 are highly conserved proteins, and that the pig proteins have greater homology with the human proteins than the mouse proteins do, suggesting that pig LGR5 and BMI1 are much more suitable references for humans.

LGR5 has been reported to accelerate proliferation in several cell lines, including skin basal carcinoma cells [25], corneal endothelial cells [26], brain cancer stem-like cells [27] and cervical cancer cells [28]. Similarly, BMI1 promotes the proliferation of normal and leukemic stem cells [29]. However, as LGR5 and BMI1 are different markers for two populations of ISCs, it was unclear whether they would have similar effects on the proliferation of intestinal epithelial cells. Therefore, in the present study, we examined IPEC-J2 cells, a pig intestinal epithelial cell line derived from the jejunal epithelia of neonatal piglets [30]. Stable strains of *LGR5*- or *BMI1*-overexpressing IPEC-J2 cells were constructed and confirmed. Due to the extremely low mRNA abundance of *LGR5* and *BMI1* in IPEC-J2 cells, knockdown or knockout experiments were not performed in the present study. Our results indicate that overexpression of either *LGR5* or *BMI1* increased the proliferation of IPEC-J2 cells.

WNT/β-catenin signaling is critical in establishing the tissue architecture during development and maintaining homeostasis in the intestinal epithelium [31]. WNT/β-catenin signaling facilitates intestinal renewal by improving the proliferation and differentiation of ISCs, a large part of which are LGR5^+^ CBCs [10,12]. However, the sensitivity of BMI1^+^ populations to WNT/β-catenin signaling perturbation is still debated. Yan et al. [6] reported that BMI1^+^ ISCs were barely sensitive to global gain- and loss-of-function WNT/β-catenin signaling modulation in vivo, whereas Cho et al. [32] demonstrated that WNT/β-catenin signaling exerted positive feedback on BMI1 expression and subsequent colony formation in MCF7 cells. The present results reveal that either *LGR5* or *BMI1* overexpression can activate WNT/β-catenin signaling and increase the protein levels of cyclin D1 and C-MYC in IPEC-J2 cells. The WNT target genes cyclin D1 and C-MYC have been confirmed as critical downstream signals promoting cell proliferation [33,34], supporting our results that *LGR5* or *BMI1* overexpression increased cell proliferation. Similarly, recent studies indicate that LGR5 and BMI1 expression in intestinal epithelia is correlated with WNT/β-catenin signaling [35,36].

To explore the effects of WNT/β-catenin signaling on cell proliferation and LGR5 and BMI1 expression, we applied rhWNT3A and XAV939. WNT3A is an activator of WNT/β-catenin signaling that has been reported to increase IEC-6 cell proliferation at a concentration of 1.5 nmol/L [37], whereas XAV939 is a small molecule inhibitor of WNT/β-catenin signaling that stimulates β-catenin degradation by stabilizing AXIN2 [38]. XAV939 has been shown to inhibit the proliferation of HEK293, SH-SY5Y and IMR-32 cells [38,39]. In the present study, the OD values in MTT assays increased when cells were supplemented with 1.5 and 3.0 nmol/L rhWNT3A. As expected, rhWNT3A activated WNT/β-catenin signaling and increased the protein levels of C-MYC, cyclin D1, LGR5 and BMI1. In addition, XAV939 supplementation suppressed WNT/β-catenin signaling and the proliferation of both *LGR5*-overexpressing and *BMI1*-overexpressing cells. Most interestingly, the inhibition of WNT/β-catenin signaling reduced the protein expression of C-MYC, cyclin D1, LGR5 and BMI1.

## 4. Materials and Methods

### 4.1. Intestinal Tissue Sample Preparation

Three male and three female 14-day-old Landrace piglets were euthanized with sodium pentobarbital before sample preparation. Their entire small intestines were then rapidly removed, washed with ice-cold phosphate-buffered saline and frozen in liquid nitrogen. All procedures were approved by the Animal Care Committee of South China Agricultural University (Guangzhou, China), 201503-027-P, 14 March 2015.

### 4.2. Cloning the cDNA of Pig LGR5

Total RNA was isolated from the piglet small intestinal mixture with Trizol reagent (Invitrogen, Carlsbad, CA, USA) in accordance with the manufacturer’s instructions. Primers were designed with Primer Premier 5.0 (Premier Biosoft International, Palo Alto, CA, USA) and synthesized by Sangon Biotech (Shanghai, China). PCR products were purified with the TIANgel Midi Purification Kit (Tiangen, Beijing, China) in accordance with the manufacturer’s protocol. Two pairs of specific primers (*LGR5A* and *LGR5B*, Table S1) were designed for PCR amplification of pig *LGR5* cDNA fragments, based on the conserved regions of the human (NM_003667.3) and mouse (NM_010195.2) sequences. The expected sequences were 1143 bp (Figure 1A) and 1727 bp (Figure 1B) long, respectively. Then, 3′ gene-specific primers (GSP2 and NGSP2, Table S1) were used to extend the cDNA end fragments by means of a SMART RACE cDNA Amplification Kit (Clontech, Palo Alto, CA, USA). Subsequently, DNAMAN 8.0 software (Lynnon BioSoft, Vaudreuil, QC, Canada) was used to align and ligate the three fragment sequences to yield the full-length cDNA sequence of pig *LGR5* containing the ORF (Figure 1D).

### 4.3. Plasmid Construction

The coding sequence of pig *LGR5* was amplified with *LGR5*-ORF primers (Table S1) containing *Bam*HI and *Xba*I (FD0055 and FD0685; Thermo Fisher Scientific, Waltham, MA, USA) enzyme digestion sites. The purified PCR product was then inserted into the *Bam*HI/*Xba*I site of the pcDNA3.1 vector (Invitrogen) to generate the *LGR5*-pcDNA3.1 recombinant plasmid. The ligation product was transferred into DH5α cells and cultured in an anti-ampicillin Luria-Bertani medium plate for 12–16 h at 37 °C. Several clones were then chosen from the plate to expand the culture, and their plasmids were extracted for PCR, enzyme digestion and sequencing identification. The plasmid was purified with the Endo-Free Plasmid Kit (Tiangen, Beijing, China) in accordance with the manufacturer’s instructions. The *BMI1*-pcDNA3.1 recombinant plasmid used in this study was constructed in our lab previously [15].

### 4.4. Cell Transfection

The basic-pcDNA3.1, *LGR5*-pcDNA3.1 and *BMI1*-pcDNA3.1 vectors were transfected into IPEC-J2 cells with Lipofectamine 3000 (Invitrogen) according to the manufacturer’s recommendations. Twenty-four hours after transfection, the cells were passaged with 0.25% trypsin-EDTA (Gibco, Carlsbad, CA, USA) at a ratio of 1:10. G418 Sulfate solution (Gibco, Grand Island, NY, USA) was added at a final concentration of 800 µg/mL. When the non-transfected cells had all died, the G418 level was reduced to 400 µg/mL. Fourteen days later, the surviving clones were picked up and the positive clones were confirmed. In the following experiments, IPEC-J2 cells transfected with basic-pcDNA3.1, *LGR5*-pcDNA3.1 and *BMI1*-pcDNA3.1 were named the control, *LGR5*-overexpressing and *BMI1*-overexpressing groups, respectively.

### 4.5. Real-Time PCR

The control, *LGR5*-overexpressing and *BMI1*-overexpressing cells were cultured in six-well plates (Corning Inc., Corning, NY, USA) at a density of 1 × 10^5^ cells/mL in growth medium for 48 h and collected for real-time PCR analysis as previously described [40]. The gene-specific primers were designed with Primer 5.0 software (Premier Biosoft International) and are detailed in Table S1. The relative expression of mRNA was calculated by the 2^−ΔΔ*C*t^ method where Δ*C*_t_ = *C*_t_ (target) − *C*_t_ (GAPDH) and ΔΔ*C*_t_ = Δ*C*_t_ (overexpressing cells) − *C*_t_ (control cells). The experiment was performed in triplicate.

### 4.6. Cells and Cell Culture

IPEC-J2 cells were maintained in growth medium (Dulbecco’s Modified Eagle’s Medium containing 10% fetal bovine serum, 50 µg/mL penicillin and 4 µg/mL streptomycin) at 37 °C in a 5% CO_2_ incubator (Shellab, Cornelius, OR, USA). The medium was changed every 48 h. The cells were passaged with 0.25% trypsin-EDTA (Gibco, Carlsbad, CA, USA) at a ratio of 1:4. WNT/β-catenin signaling activity was activated by the addition of rhWNT3A (R&D Systems, Minneapolis, MN, USA) or inhibited by the addition of XAV939 (Selleckchem, Houston, TX, USA) to the growth medium.

### 4.7. Cell Proliferation Analysis

Cell proliferation was determined by the cell count and MTT assays, as previously described [41,42].

Cell count assay. Cells were cultured in six-well plates (Corning) at a density of 1 × 10^5^ cells/mL and counted following trypan blue staining. Briefly, cells were detached with 0.25% trypsin (Sigma, St. Louis, MO, USA) for 10 min at 37 °C after being washed with phosphate-buffered saline, and then were blocked with an equal volume of growth medium. Viable cells were counted with a hemocytometer under an Inversion Microscope System (Nikon, Gotenba, Japan). The experiment was performed in triplicate.

MTT assay. Cells were seeded in 96-well plates (Corning) at a density of 1 × 10^4^ cells/mL in growth medium. Twenty microliters of 5 mg/mL 3-(4,5-dimethylthiazol-2-yl)-2, 5-diphenyltetrazolium bromide (MTT) solution (Sigma) was added to each well and incubated for 4 h. After the supernatant was carefully removed, 150 μL of dimethyl sulfoxide was added to each well. The plate was slightly shaken for 30 min at room temperature. The OD values of the products were measured on a microplate reader at a wavelength of 490 nm. The experiment was performed in triplicate.

### 4.8. Western Blot

Cells were cultured in six-well plates (Corning) at a density of 2 × 10^5^ cells/mL in growth medium. At 72 h after seeding or 48 h after treatment, the cells were collected for Western blot analysis as previously described [43]. Briefly, the cell lysates were separated by sodium dodecyl sulfate polyacrylamide gel electrophoresis and transferred onto polyvinylidene difluoride membranes. After being blocked with 5% fat-free milk, the membranes were incubated at 4 °C overnight with primary antibodies, and then for 2 h with secondary antibodies. The proteins were visualized with an ECL Plus chemiluminescence detection kit (Beyotime, Shanghai, China) on a FluorChem M system (Cell Biosciences, San Leandro, CA, USA). The density of the bands was analyzed with Image J Software (version 1.8.0_112, National Institute of Health, Bethesda, MD, USA). The experiment was performed in triplicate.

The antibodies used in this study were as follows: anti-C-MYC (#5605) and anti-β-actin (#4970) from Cell Signaling Technology (Beverly, MA, USA); anti-phospho-β-catenin (Ser33) (sc-16743), anti-TCF4 (sc-13027), anti-GSK3β (sc-9166), anti-cyclin D1 (sc-753) and anti-BMI1 (sc-10745) from Santa Cruz (Dallas, TX, USA); anti-β-catenin (ab6302) and anti-AXIN2 (ab109307) from Abcam (Cambridge, MA, USA); anti-LGR5 from ABGENT (San Diego, CA, USA); and anti-rabbit IgG and anti-mouse IgG from Beijing Biosynthesis Biotechnology (Beijing, China). RhWNT3A was purchased from R&D Systems (Minneapolis, MN, USA), and XAV939 was purchased from Selleckchem (Houston, TX, USA).

### 4.9. Statistical Analysis

The data were processed with SPSS software version 19.0 (SPSS Inc., Chicago, IL, USA). Variance analysis was performed with Student’s *t*-test or one-way analysis of variance followed by a Least Significant Difference test. Differences between groups were considered statistically significant if *p* < 0.05. Data are expressed as the mean ± standard error (SE).

## 5. Conclusions

In conclusion, we are the first group to clone the cDNA containing the full-length ORF of pig *LGR5*. Overexpression of either *LGR5* or *BMI1* promotes cell proliferation and WNT/β-catenin signaling. Moreover, activating WNT/β-catenin signaling promotes cell proliferation and LGR5 and BMI1 protein expression, while inhibiting it has the opposite effects. According to the current available data, LGR5 and BMI1 increase the proliferation of pig intestinal epithelial cells by stimulating WNT/β-catenin signaling. However, further studies will be valuable in confirming these results.

## Supplementary Materials

Supplementary materials can be found at http://www.mdpi.com/1422-0067/19/4/1036/s1.

## Abbreviations

APC	adenomatous polyposis coli
BMI1	B-cell-specific Moloney murine leukemia virus insertion site 1
CBC	crypt base columnar
GSK3β	glycogen synthase kinase 3β
IPEC-J2	pig intestinal epithelial cells
ISC	intestinal stem cell
LGR5	Leucine-rich repeat-containing G protein-coupled receptor 5
OD	optical density
ORF	open reading frame
PTEN	phosphatase and tensin homolog
TCF	T-cell factor
