# Supplementary material for: LGR5 and BMI1 Increase Pig Intestinal Epithelial Cell Proliferation by Stimulating WNT/β-Catenin Signaling
Source: Int J Mol Sci. 2018 Mar 30;19(4):1036. doi: 10.3390/ijms19041036 (PMC5979389; doi:10.3390/ijms19041036)
Supplement: Supplementary file 1 [file ijms-19-01036-s001.pdf]

Table S1. Primers used for cloning the *LGR5* CDS and real-time PCR.

Gene	Primer sequence (5′-3′)	Product size (bp)
GSP2	CCCTTTGCTTCCTGGTGATG	
NGSP2	GGCTTTCTTGTCCTTCTCCTCTT	
LGR5A	F: TGACGACGAGGAAGACCTGAAAGCCCTACA<br>R: TTAGAGACATGGGACAAATGCCACGGAAGAGAG	1143
LGR5B	F: ATGGACACCTCCTCGGTTGGCGTGCTC<br>R: GTGCCGCAAGTAAGTGCCAAAACTGCTATG	1727
LGR5 (ORF)	F: CGGGATCCATGGACACCTCCTCGG<br>R: GCTCTAGATTAGAGACATGGGACAAATGCCACGGA	2740
LGR5 (RT)	F: GCTGGCTGCCGTGGATGC<br>R: AGCAGGGCGCAGAGGACAAG	237
BMI1	F: AAGGAGAAATCTAAGGAGGA<br>R: CCAGGTATAAATGTAGGCAA	195
GAPDH	F: AGGTCGGAGTGAACGGA<br>R: TGGGTGGAATCATACTGG	145

GSP2 = 3′ RACE out primer; NGSP2 = 3′ RACE nested primer; *LGR5A* = primers for cloning *LGR5* A fragment; *LGR5B* = primers for cloning *Lgr5* B fragment; *LGR5* (ORF) = primers for cloning *Lgr5* ORF; *LGR5* (RT) = primers for real-time PCR of *LGR5*; *BMI1* = primers for real-time PCR of *BMI1*; *GAPDH* = glyceraldehyde-3-phosphate dehydrogenase; the underlined sequences in the F and R primers are the enzyme digest sites of *Bam*HI and *Xba*I, respectively.
